# An Overview of Vaccination Strategies and Antigen Delivery Systems for *Streptococcus agalactiae* Vaccines in Nile Tilapia (*Oreochromis niloticus*)

**DOI:** 10.3390/vaccines4040048

**Published:** 2016-12-13

**Authors:** Hetron Mweemba Munang’andu, Joydeb Paul, Øystein Evensen

**Affiliations:** Section of Aquatic Medicine and Nutrition, Department of Basic Sciences and Aquatic Medicine, Faculty of Veterinary Medicine and Biosciences, Norwegian University of Life Sciences, Adamstuen Campus, Ullevålsveien 72, P.O. Box 8146, Dep NO-0033, Oslo 0454, Norway; joydeb.paul@nmbu.no (J.P.); oystein.evensen@nmbu.no (Ø.E.)

**Keywords:** *Streptococcus agalactiae*, vaccines, tilapia, antigen, dose, DNA, subunit, challenge

## Abstract

*Streptococcus agalactiae* is an emerging infectious disease adversely affecting Nile tilapia (*Niloticus oreochromis*) production in aquaculture. Research carried out in the last decade has focused on developing protective vaccines using different strategies, although no review has been carried out to evaluate the efficacy of these strategies. The purpose of this review is to provide a synopsis of vaccination strategies and antigen delivery systems currently used for *S. agalactiae* vaccines in tilapia. Furthermore, as shown herein, current vaccine designs include the use of replicative antigen delivery systems, such as attenuated virulent strains, heterologous vectors and DNA vaccines, while non-replicative vaccines include the inactivated whole cell (IWC) and subunit vaccines encoding different *S. agalactiae* immunogenic proteins. Intraperitoneal vaccination is the most widely used immunization strategy, although immersion, spray and oral vaccines have also been tried with variable success. Vaccine efficacy is mostly evaluated by use of the intraperitoneal challenge model aimed at evaluating the relative percent survival (RPS) of vaccinated fish. The major limitation with this approach is that it lacks the ability to elucidate the mechanism of vaccine protection at portals of bacterial entry in mucosal organs and prevention of pathology in target organs. Despite this, indications are that the correlates of vaccine protection can be established based on antibody responses and antigen dose, although these parameters require optimization before they can become an integral part of routine vaccine production. Nevertheless, this review shows that different approaches can be used to produce protective vaccines against *S. agalactiae* in tilapia although there is a need to optimize the measures of vaccine efficacy.

## 1. Introduction

Tilapia culture has surged to become one of the leading farmed fish species in the world since the 1990s with commercial production capacity of 2.5 million tons in 2007 [1]. By 2008, it ranked fifth and by 2014 it was highest, reaching >3.5 million tons after carp (*Cyprinus carpio*) and salmon (*Salmo salar* L.) [1]. This rapid expansion has brought with it an upsurge in the number of diseases infecting tilapia because of the intensified farming systems used, leading to high stocking densities aimed at increasing production outputs. High stocking densities induce stress-related immunosuppression, rendering fish to be highly susceptible to disease infections [2], and increase the transmission index of infectious pathogens in cultured fish [2]. One of the important diseases that has plagued tilapia production is streptococcosis, caused by *Streptococcus agalactiae* infection. The symptoms caused by this disease include septicemia, anorexia, exophthalmia, corneal opacity and ascites, leading to high mortalities in infected fish [3]. Its devastating impact on tilapia production has led to increased antibiotics and other drugs usage, which has raised serious concerns on environmental drug release [4,5]. The most environmentally friendly disease control strategy is vaccination. As such, the search for protective vaccines against *S. agalactiae* has significantly intensified alongside the rapid expansion of tilapia production in the last two decades.

Given that *S. agalactiae* is an emerging disease in tilapia, a fish species whose production capacity has only increased to global markets in recent years, there are several factors in vaccine production that require optimization. These include the need for a comprehensive understanding of the infection biology of the disease in tilapia in order to pave the way into elucidating the immunological mechanisms by which vaccination confers protection. It is not clear whether there is a standardized challenge model that can be applied across different vaccination trials in order to compare the efficacy of different vaccine batches. Moreover, the measures of vaccine efficacy have not been clearly defined as to whether antibodies can be used as a measure of protective immunity or relative percent survival (RPS) is the gold standard for measuring the protective ability of *S. agalactiae* vaccines in tilapia. Moreover, the bacteria has several proteins able to serve as vaccine antigens, which has attracted a lot of interest in the design of subunit and DNA vaccines. The challenge has been to identify the most immunogenic protein, able to confer the highest protection in vaccinated fish. Despite these knowledge gaps, the search for protective vaccines against *S. agalactiae* in tilapia has continued. However, it has become apparent that there is a need to evaluate the immunization strategies and vaccine designs currently in use in order to identify some of the factors that have derailed our success in developing protective vaccines against *S. agalactiae* in tilapia.

Hence, this review brings into perspective different antigen delivery systems used in the design of *S. agalactiae* vaccines, as well as the different immunization strategies used to administer vaccines in tilapia. In addition, it brings into perspective the different methods currently used for evaluating the efficacy of *S. agalactiae* vaccines in tilapia. Lastly, it explores the possibility of developing correlates of vaccine protection, based on existing data, that could serve as benchmarks for optimizing newly developed vaccines against *S. agalactiae* in tilapia.

## 2. Antigen Delivery System

The antigen delivery systems used for the design of *S. agalactiae* vaccines in tilapia can be classified into replicative and non-replicative vaccines.

### 2.1. Replicative Antigen Delivery Systems

Replicative antigen delivery systems used for the design of *S. agalactiae* vaccine for tilapia comprise of live attenuated, heterologous live vector and DNA vaccines [6,7].

#### 2.1.1. Live Attenuated Vaccines

Thus far, two approaches have been used to attenuate virulent trains of *S. agalactaie* into avirulent strains for use as live vaccines in tilapia. These include (i) serial passaging [8], and (ii) chemical treatment [9]. Pridgeon and Klesius [9] attenuated different isolates of *S. agalactiae* by treating them with sparfloxacin. When strains that developed resistance to sparfloxacin were tested for pathogenicity in tilapia, they were found to be avirulent and were highly protective (RPS = 100%) against challenge using highly pathogenic strains of *S. agalactiae*. All avirulent strains lost their hemolytic property, suggesting that hemolysis was linked to pathogenicity of *S. agalactiae* in tilapia. In another study, Pridgeon et al. [10] attenuated *S. agalactiae* by selecting for resistance to gossypol, proflavine hemisultate, novobiocin and ciprofloxacin. Although not all chemicals were effective, novobiocin attenuated some of the pathogenic strains into avirulent strains that became highly protective when used as live vaccines in tilapia. Overall, these studies show that selecting for drug resistance can be an effective strategy for generating avirulent strains able to serve as live vaccines against *S. agalactiae* in tilapia.

Another approach used for generating avirulent strains is serial passage of pathogenic strains on growth media. Wang et al. [11] showed that serial passaging of pathogenic strains of *S. agalactiae* led to deletions of virulence factors culminating in the production of avirulent strains that were highly protective when used as a live vaccine in tilapia. Similarly, Li et al. [8] showed that a serially passaged pathogenic strain of *S. agalactiae* lost its virulence and when injected as a live vaccine at 10^9^ CFU/fish and orally administered at 10^10^ CFU/fish showed high protection without causing clinical disease in vaccinated fish. Overall, Table 1 and Table 2 show that live attenuated vaccines are more protective than inactivated vaccines.

Although reversion of avirulent strains to virulence has not been demonstrated for *S. agalactiae* live vaccines used in aquaculture, studies carried out for some fish pathogens have shown reversion of avirulent strains to virulence in infected fish subjected to stress [20]. Hence, it is likely that there are environmental and fish biological factors that could lead to reversion of avirulent strains to virulence of *S. agalactiae* live vaccines. Moreover, it is also feared that fish vaccinated with live vaccines could become a source of infection to other aquatic organisms in which the avirulent vaccine strain could be pathogenic. Hence, it is important that these factors are taken into consideration before the live vaccines developed under experimental conditions can be considered for commercial use.

#### 2.1.2. DNA Vaccines

DNA vaccines have been made using different surface proteins of *S. agalactiae* encoded in plasmid vectors [12,13]. Huang et al. [12] used the surface immunogenic protein (Sip) while Nur-Nazifah et al. [13] used the LPXT motif cell wall surface anchor family protein that produced high protection in vaccinated fish [21]. Apart from these proteins, Table 3 shows other proteins of *A. agalactiae* that could serve as vaccine candidates for use in DNA vaccine production. It is interesting to note that the DNA vaccines made by Huang et al. [12] and Nu-Nazifah et al. [13] showed higher protection than most of the inactivated whole cell (IWC) vaccines shown in Table 2 suggesting that Sip and LXPT proteins encoded in these DNA vaccine were highly potent protective antigens.

#### 2.1.3. Heterologous Live Vector Vaccines

Heterologous live vector vaccines are replicative vaccines encoding the antigenic protein of a virulent pathogen inserted in another replicative organisms that does not cause disease in the target host to be vaccinated [29]. Huang et al. [12] inserted the Sip protein of *S. agalactiae* into the *Salmonella typhimurium* plasmid vector and used it as a live vaccine against *S. agalactiae* in tilapia. The objective of using the *S. typhimurium* live vector is that it is not pathogenic in tilapia, but is able to replicate in order to continuously produce the encoded Sip protein in vaccinated fish as a live vaccine. Apart from being an antigen carrier, a live heterologous vector can also serve as an adjuvant able to activate antigen presenting cells (APCs) to enhance antigens uptake, processing and presentation to cells of the adaptive immune system [29].

### 2.2. None-Replicative Antigen Delivery System

Non-replicative antigen delivery systems used for the design of *S. agalactiae* vaccines for tilapia include the inactivated whole cell (IWC), subunit and extracellular vaccines.

#### 2.2.1. Whole Cell Inactivated Vaccines

As shown in Table 2, IWC vaccines account for the largest proportion of vaccines developed against *S. agalactiae* in tilapia. These vaccines are considered safe because they are “killed” and as such, they pose no danger of reversion to virulence [6]. They require the help of adjuvants to enhance their immunogenicity [7,30]. Different adjuvants have been used in IWC-vaccine formulations for *S. agalactiae* in tilapia and these include Freund’s incomplete [22,28] and aluminum hydroxide gel (AH) [22] adjuvants. However, adjuvants have been shown to induce side effects in fish [31] and, hence, the search for other antigen delivery systems that do not require incorporation of adjuvants in vaccine formulation has attracted a lot of interest in fish vaccinology [7].

#### 2.2.2. Subunit Vaccines

Subunit vaccines are none-replicative vaccines designed to encode serotype specific or ubiquitous antigenic proteins designed to cover a broad range of serotypes. For example, the Sip protein is a cross protective protein for different strains of *S. agalactiae* [32]. It is highly conserved, having more than 90% sequence alignment homology between tilapia and mammalian strains of *S. agalactiae* [33]. He et al. [22] produced a subunit vaccine using the Sip protein that produced high protection in tilapia while Yi et al. [23] showed high protection in tilapia using the subunit vaccines encoding the fibrinogen binding protein A (FbsA) and α-enolase antigens. Similarly, Wang et al. [24] produced a subunit vaccine against *S. agalactiae* using the phosphoglycerate kinase (PGK) protein expressed in the *Escherichia coli* plasmid and showed that combining PGK with IWC-vaccines significantly increased the survival of tilapia against *S. agalactiae* infection. In addition, PGK increased the expression of IL-1β and TNFα suggesting that this protein evoked the proinflammatory cytokine responses in order to enhance the uptake of IWC-antigens in vaccinated fish. Table 4 shows some of the subunit vaccines developed under experimental studies while Table 3 shows the immunogenic proteins identified as potential antigens for subunit vaccines.

#### 2.2.3. Extracellular Products

Bacteria extracellular proteins (ECPs) have been shown to produce protective immunity against different fish diseases [15,35]. Pasnik et al. [15] produced ECPs by concentrating the cell free-fluid obtained after *S. agalactiae* culture in tryptose soy broth (TSB) at 27 °C for 72–125 h. The ECP vaccine was prepared by concentrating the cell free fluid followed by 3% formalin inactivation. Immunization using the ECP vaccine showed low protection against *S. agalactiae* challenge in tilapia. However, when the ECP vaccine was combined with the IWC-vaccine, it produced higher protection than the IWC-vaccine without ECPs, indicating that the highest protection was only attained when both the IWC and ECP vaccines were used together. Given that ECPs are chemotactic [36], it is likely that their role was to attract APCs to sites of IWC-antigen deposition for enhanced antigen uptake while the IWC-antigens induced adaptive immune responses once they were presented to the B- and T-lymphocytes by APCs. Put together, these observations indicate that a combined input of ECPs and IWC-antigens is required to produce long-term protective immunity for *S. agalactiae* vaccines in tilapia.

## 3. Vaccine Delivery System

Vaccine delivery systems used for administering *S. agalactiae* vaccines in tilapia can be divided into parenteral and mucosal vaccination.

### 3.1. Parenteral Vaccination

Intraperitoneal and intramuscular injection are the only parenteral routes explored for administering *Streptococcus* vaccines in tilapia as shown below.

#### 3.1.1. Intraperitoneal Injectable Vaccines

Table 1, Table 2 and Table 4 show that intraperitoneal injectable vaccines account for the largest proportion of vaccines developed against *S. agalactiae* in tilapia and the general observation is that injectable vaccines produce higher protection than mucosal vaccines [37,38]. The advantage of using injectable vaccines is that it is easy to optimize the antigen dose that corresponds with protection, thereby making it is easy to correlate the antigen dose with vaccine protection [39]. This can be attributed to the fact that vaccines administered inside the fish by injection are quickly taken up for the induction of adaptive immune responses, unlike mucosal vaccines that have to cross-mucosal barriers before gaining entry into the systemic environment. However, the disadvantage of injectable vaccines is that they demand individual handling of fish, which could lead to handling-related mortalities and stress-related immunosuppression. Hence, there is a need to develop vaccination strategies that are less stressful to fish but have the ability to produce long-term protective immunity.

#### 3.1.2. Intramuscular Injectable Vaccines

In fish, this mode of vaccine delivery is mostly used for administering DNA vaccines [30,40] that require replication at the injection sites before uptake of antigens by antigen presenting cells (APCs). In tilapia, it has been explored for the DNA vaccines used against *S. innae* [41] although its efficacy for *S. agalactiae* DNA vaccines is yet to be determined. Ramadan et al. [42] showed higher protection for *Aeromonas hydrophila* intramuscular vaccines compared to immersion vaccines in tilapia. This can be attributed to the fact that intramuscular vaccines gain quick entry into the systemic environment, which enhances their ability to evoke adaptive immune responses unlike immersion vaccines whose antigens have to cross mucosal barriers before entering the systemic environment.

### 3.2. Mucosal Vaccination

Mucosal immunization strategies explored for administering *S. agalactiae* vaccines in tilapia include immersion, spray and oral vaccination as shown below.

#### 3.2.1. Bath/Immersion Vaccination

Table 1, Table 2 and Table 4 show that immersion vaccines account for a small proportion of vaccines used against *S. agalactiae* compared to injectable vaccines, in tilapia. Although this vaccination strategy is less used compared to the injectable vaccines, its main advantage is that it is cost effective because a large number of fish can be vaccinated at the same time without individual handling. As such, it is less likely to cause mortality during vaccination and it impacts less stress-related immunosuppression.

#### 3.2.2. Spray Vaccination

Noraini et al. [19] developed a formalin IWC vaccine that was administered by spraying fish on the skin surface. The vaccine produced high protection in vaccinated fish after intraperitoneal (RPS = 70%) and immersion (RPS = 80%) challenge. This mode of vaccination has only been reported in one study [19] and, hence, there is a need for more vaccine efficacy trials using spray vaccination to validate its consistency in protecting fish against *S. agalactiae* infection.

#### 3.2.3. Oral Vaccination

Both live attenuated and DNA vaccines have been used for oral immunization of tilapia against *S. agalactiae* (Table 1). Nur-Nazifah et al. [13] compared the efficacy of a recombinant vaccine encoding the LPXT cell wall surface anchor family protein of *S. agalactiae* with an IWC-vaccine in which they showed that both vaccines produced high IgM levels in serum and mucus that corresponded with low post challenge infection and high RPS in vaccinated fish. Mohd et al. [43] showed high protection in tilapia orally vaccinated using an IWC-vaccines while Huang et al. [12] used a live recombinant *S. typhimurium* vector vaccine encoding the Sip protein and produced high protection levels against *S. agalactie*. These studies show that both live and inactivated vaccines can produce high protection levels when orally administered in tilapia.

Zhang et al. [34] pointed out that conventional oral vaccines with simple architecture face barriers with regard to stimulating effective mucosal immunity. To overcome this problem, they developed a phase-transitional shielding layer made of a poly[(methyl methacrylate)-co-(methyl acrylate)-co-(methacrylic acid)]-poly(d,l-lactide-co-glycolide) (PMMMA-PLGA), able to protect the vaccine antigens in the digestive tract in order to achieve targeted immune responses in the intestine. Tilapia vaccinated using the Sip antigen encapsulated in the PMMMA-PLGA nanoparticles showed high protection against *S. agalactaie* that lasted for several months. Evans et al. [16] compared the efficacy of an oral vaccine with an injectable vaccine made from the same batch and showed that the injectable vaccine induced higher protection (RPS = 70%) than the oral vaccine (RPS = 25%), which is in agreement with observations made by other scientists that injectable vaccines produce higher protection levels than mucosal vaccines [38].

## 4. Measures of Vaccine Efficacy

Establishing the measures of vaccine efficacy is an integral part of vaccine development in order to ensure that the vaccines produced are protective. This involves several steps, such as (i) developing a reliable and reproducible challenge model, (ii) establishing the measures of vaccine protection, and (iii) establishing the correlates of protective immunity able to serve as benchmarks for the optimization of newly developed vaccines.

### 4.1. Challenge Models

As pointed out from our previous studies [44], factors that constitute a reproducible challenge model include (i) the use of a highly virulent bacterial strains able to cause high mortality in susceptible fish; (ii) optimization of the challenge dose; (iii) use of highly susceptible fish able to produce a wide discriminatory capacity between the vaccine protected fish and the unvaccinated control fish; (iv) use of an infection method that mimics natural disease transmission; and (v) estimation of sample size able to show significant statistical differences between the vaccinated and control fish. Thus far, there is no challenge model developed encompassing all these attributes for evaluating the efficacy of *S. agalactiae* vaccines in tilapia.

As shown in Table 1, Table 2 and Table 4, the most widely used challenge model for evaluating vaccine efficacy in tilapia is by intraperitoneal injection of pathogenic strains of *S. agalactiae* in vaccinated fish. Deposition of bacteria by injection in fish enables the bacteria to gain quick entry into the systemic environment where it spreads to internal organs in a short time. The major limitation to this mode of challenge is that it does not mimic natural infection because the bacteria do not gain entry through the natural portals of entry via mucosal surfaces into the systemic environment. On the contrary, challenge by bath, immersion or cohabitation mimics the nature of the infection by enabling bacteria to colonize and penetrate mucosal surfaces before gaining entry into the systemic environment. As a result, challenge via mucosal routes is likely to give a better understanding of the sequential progression of infection, starting from bacteria colonization at portals of entry on mucosal surfaces followed by bacterial penetration and onward dissemination to internal organs. Hence, challenge through mucosal surfaces would pave the way into elucidating the mechanisms of vaccine protection by showing the ability of vaccinated fish to prevent bacteria colonization on mucosal surfaces, blocking bacteria penetration into the systemic environment and preventing tissue damage in target organs. Therefore, there is a need to develop effective challenge models, able to provide a detailed understanding of the mechanisms of vaccine protection alongside overall protection against lethal challenge.

### 4.2. Measure of Vaccine Protection

The measures of protection explored thus far for *S. agalactiae* vaccines in tilapia include RPS, bacteria quantification by quantitative PCR (qPCR), Serum inhibition test (SIT) and prevention of pathology as shown below.

#### 4.2.1. Relative Percent Survival

Table 1, Table 2 and Table 4 show that RPS is the most widely used approach to evaluate the efficacy of *S. agalactiae* vaccines in tilapia. This is based on determining the number of vaccinated fish that survive lethal challenge against pathogenic strains of *S. agalactiae* infection relative to the number of the unvaccinated control fish. The major limitation with this measure of efficacy is that it only determines the survival rate against mortality, but it does not give detail on the mechanisms of vaccine protection in vaccinated fish. It does not show whether vaccine induced protection prevents the colonization of bacteria on mucosal surfaces, prevents dissemination of bacteria to internal organs or blocks tissue damage in the target organs of vaccinated fish. Hence, there is a need for non-lethal measures of vaccine efficacy that show the mechanisms of vaccine protection apart from determining RPS after lethal challenge.

#### 4.2.2. Bacterial Quantification Using Quantitative PCR

Su et al. [3] developed a quantitative PCR assay whose primer pair IGS-s/IGS-a targets the 16-23S rRNA intergenic spacer region of *S. agalactiae* for monitoring bacteria colonization on mucosal surfaces and tissue tropism. In their study [3], they showed that bacteria loads were highest in the brain followed by the kidney, heart, spleen, intestines and eyes. Tissues with low bacterial loads included the muscle, liver and gills. Given that *S. agalactiae* replicates in the blood stream and that it has the capacity to cross the brain barrier [45], the qPCR developed by Sun et al. [3] can be used to establish the optimal levels of vaccine induced protection required to prevent bacteria replication in the blood and to prevent the entry of bacteria in the brain and other target organs. Moreover, it can also be used to determine the optimal levels of vaccine-induced protection to prevent bacteria colonization on mucosal surfaces and penetration into the systemic environment of vaccinated fish.

#### 4.2.3. Serum Inhibition Test

The serum inhibition test (SIT) is a method used to measure the ability of antibodies from vaccinated fish to inhibit bacteria growth in vitro. Maiti et al. [46] used the SIT to evaluate the ability of antibodies induced by an outer membrane protein A (OmpA) vaccine in carp (*Cyprinus carpio*) to inhibit *Edwardsiella tarda* growth in vitro while Rauta and Nayak [47] used the SIT to determine the ability of antibodies from rohu (*Labeo rohita*) vaccinated using a recombinant OmpA vaccine to inhibit *Aeromonas hydrophila* growth in vitro. Dubey et al. [48], used the SIT to show the ability of antibodies produced by a recombinant OmpA antigen encapsulated in chitosan nanoparticles in rohu to inhibit *A. hydrophila* growth in vitro in a dose-dependent manner in which fish orally vaccinated with a high antigen dose (HiAg) (8 μg/g) showed a higher inhibition capacity of *A. hydrophila* than fish vaccinated with a low antigen (LoAg) (4 μg/g) dosage vaccine, which corresponded with post challenge survival proportions in which the HiAg dose group showed a higher RPS (79.99%) than the LoAg dose (RPS = 37.33%), demonstrating that the SIT can be used as an in vitro measure of vaccine efficacy, able to determine the protective ability of antibodies generated by vaccination. In tilapia, Zhang et al. [34] used the SIT to evaluate the ability of antibodies induced by the Sip protein administered using the Poly[(methyl methacrylate)-co-(methyl acrylate)-co-(methacrylic acid)]-poly(d,l-lactide-co-glycolide) (PMMMA-PLGA) to inhibit *S. agalactiae* replication in vitro. Therefore, the SITs can be used as a measure of vaccine efficacy to determine bacteria inhibition capacity of serum obtained from vaccinated fish.

#### 4.2.4. Prevention of Pathology

Su et al. [3] showed that an increase of bacterial titers corresponded with the establishment of pathology leading to meningitis, hemorrhages in the eyes, exophthalmia and corneal opacity in tilapia infected with *S. agalactiae*. Therefore, bacteria quantification can be optimized to determine the cutoff limit that corresponds with the establishment of tissue damage in target organs, which could serve as a correlate of pathology. For example, in our previous studies, we showed that viral loads >1 × 10^7^ TCID_50_/mL in Atlantic salmon (*Salmo salar* L.) infected with infectious pancreatic necrosis virus (IPNV) correlated with the establishment of pathology in the target organs. Hence, this titer served as a correlate of pathology, thereby serving as a diagnostic marker of tissue damage in target organs. On the other hand, we carried out different studies that showed that antibody titers >1.4 OD_490_ (diluted at 1:50) prevented the establishment of pathology linked to RPS >90% in vaccinated fish [39,49,50]. Hence, while a viral load ≥1 × 10^6.5^ TCID_50_/mL could serve as a correlate of pathology, antibody titers >1.4 OD_490_ (diluted at 1:50) could serve as a correlate of protection against pathology and RPS >90% in vaccinated fish [39]. Using a similar approach, vaccine development can be optimized to produce antibody levels that correlate with the prevention of pathology as a measure of efficacy in fish vaccinated against *S. agalactiae*.

### 4.3. Correlates of Protective Immunity

Studies carried out in higher vertebrates have shown that antibodies are the most widely used correlates of protection for the licensure of vaccines in mammals [51,52]. Similar correlates of protection can be developed for *S. agalactiae* vaccines for tilapia to serve as benchmarks in the optimization of newly developed vaccines as discussed below.

#### 4.3.1. Antibody Levels as Correlates of Protective Immunity for *S. agalactiae* in Tilapia

Prasnik et al. [14], showed a high correlation between RPS and antibody titers in tilapia vaccinated against *S. agalactiae*. In their study, they vaccinated Nile tilapia using an IWC vaccine and showed significantly high correlations of *r*^2^ = 0.792 (*p* = 0.0004) and *r*^2^ = 0.883 (*p* = 0.00) between antibody responses and RPS at 47 and 180 days post challenge (dpc), respectively. These findings demonstrate that antibody responses can be correlated with vaccine protection in tilapia vaccinated against *S. agalactiae*, which is in agreement with our previous findings [39] in which we showed that antibodies could serve as correlates of protective immunity in vaccinated fish. Hence, an antibody titer that correlates with the prevention of mortality in tilapia vaccinated against *S. agalactiae* can be developed to serve as a benchmark for the optimization of newly developed vaccines.

#### 4.3.2. Antigen Dose as A Correlate of Protection for *S. agalactiae* Vaccines in Tilapia

In our previous studies, we have shown that antigen dose can be optimized to correspond with vaccine protection in vaccinated fish [39,48]. Similarly, Li et al. [8] showed an antigen dose-dependent immune protection in fish vaccinated with vaccine doses of 10^5^, 10^6^, 10^7^, 10^8^ and 10^9^ CFU/fish that corresponded with increasing RPS = 10.15%, 35.48%, 50.74%, 64.52% and 67.74% for fish challenged at 15 days post vaccination, respectively. In another study, Huang et al. [53] showed a dose-dependent immune protection in which fish vaccinated with antigen doses of 10^7^, 10^8^ and 10^9^ CFU/fish showed corresponding increasing levels of RPS = 20%, 27% and 33% after a single vaccination dose. For fish immunized twice using the same antigen doses, protection correspondingly increased to RPS = 30%, 40% and 47% while fish immunized thrice showed a further corresponding increase of RPS = 47%, 53% and 57%, respectively. These findings show that the antigen doses used to vaccinate tilapia against *S. agalactiae* corresponded with post challenge protection levels. These findings also show that increasing the number booster vaccinations using the same antigen doses further increased the post challenge survival of vaccinated fish. In summary, these findings demonstrate that a correlate of vaccine protection against mortality can be established based on antigen dose and that a homologous prime-boost vaccination regime can also be established that confers the highest protection in vaccinated fish.

## 5. Genotype and Biotype Diversity as a Challenge to Vaccine Efficacy

Several studies have shown that *S. agalactiae* exists as variant strains classified into different biotypes. As a result, different methods of characterizing different biotypes and strains of *S. agalactiae* have been developed, such as the traditional serotyping, molecular serotyping, ISR-SSCP, AFLP, MLST and PFGE [16,21,54,55,56,57,58,59]. However, it is not clear as to whether these strains are cross protective. Paul [60] compared the cross reactive ability of antibodies generated from fish vaccinated against *S. agalactiae* biotypes I and II when tested against their homologous and heterologous antigens. The findings obtained from this study showed that fish vaccinated against biotype I had higher antibody titers when tested against its homologous and heterologous antigen than antibodies generated from fish vaccinated using the biotype II vaccine. The major limitation with this study is that there was no challenge done in order to determine the protective ability of these vaccines against challenge using their homologous and heterologous bacterial strains. Hence, there is a need for further studies to demonstrate the cross protective ability of different strains of *S. agalactiae* using vaccination and challenge studies.

## 6. Conclusions

This review has shown that both replicative and non-replicative antigen delivery systems have been used in the design of *S. agalactiae* vaccines in tilapia. The replicative antigens delivery systems explored include the live attenuated avirulent strains, heterologous live vector and DNA vaccines, while the non-replicative vaccines include IWC, ECPs and subunit vaccines. In general, IWC vaccines account for the largest proportion of vaccines explored for *S. agalactiae* in tilapia, although the general observation is that replicative vaccines produce higher protection than the non-replicative vaccines. Immunization strategies explored thus far include intraperitoneal vaccination, which accounts for the largest of proportion of vaccine delivery systems used in tilapia. Oral vaccination accounts for the largest proportion of the mucosal vaccine delivery systems, unlike the spray and immersion vaccines, which have been less studied for the vaccination of tilapia against *S. agalactiae*.

The most widely used challenge model for evaluating the efficacy of *S. agalactiae* vaccines in tilapia is the intraperitoneal injection of pathogenic strains in vaccinated fish followed by determining the RPS [61]. The major limitation with this challenge model is that it precludes the ability to evaluate the mechanisms of vaccine protection at portals of bacteria entry into mucosal organs because the challenge bacteria are forcibly introduced inside the fish by injection. Future studies should seek to develop challenge models that mimic natural infection to pave the way in elucidating the mechanisms of vaccine protection during infection progression starting from the prevention of bacteria colonization on mucosal surfaces, penetration into the systemic environment, dissemination and the establishment of pathology in target organs. Despite this, indications are that the correlates of vaccine protection can be established based on antibody responses and antigen dose. Overall, this review shows that different approaches can be used to develop protective vaccines against *S. agalactiae* in tilapia although there is a need to optimize the measures of vaccine efficacy.

## Figures and Tables

**Table 1:** Live attenuated and DNA vaccines showing delivery systems and modalities of vaccination.

Vaccine	Vaccination	Challenge	RPS *	Ref.
Live attenuated vaccination	Intraperitoneal	Intraperitoneal	70%	[10]
Live attenuated vaccination (YM001)	Intraperitonal	Intraperitneal	96.88%	[8]
Live attenuated vaccination (YM001)	Immersion	intracoelomic	67.22%	[8]
Live attenuated vaccination (YM001)	Oral	Intraperitoneal	71.81%	[8]
Live attenuated vaccination	Intraperitoneal	Intraperitoneal	75%–100%	[9]
DNA vaccine (Sip) *Salmonella typhimurium* vector	Oral	Intraperitoneal	70%–100%	[12]
Recombinant DNA feed based vaccine	Oral	Intraperitoneal	70%	[13]

* RPS = relative percent survival.

**Table 2:** Inactivated whole cell vaccines showing delivery systems and modalities of vaccination.

Vaccine	Vaccination	Challenge	RPS	Ref.
Whole cell Inactivated vaccine—formalin killed	Intraperitneal	Intraperitneal	49%	[14]
Whole cell Inactivated vaccine—formalin killed	Intraperitneal	Intraperitneal	50%	[15]
Whole cell Inactivated vaccine—formalin killed	Intraperitneal	Intraperitneal	80%	[16]
Whole cell Inactivated vaccine—formalin killed	Bath	Intraperitneal	34%	[16,17]
Whole cell Inactivated vaccine—formalin killed	Oral	Intraperitneal	97%	[16,17]
Whole cell inactivated vaccines—heat killed	Oral	intracoelomic	38.9%	[18]
Whole cell Inactivated vaccine—formalin killed	Spray	Immersion	80%	[19]
Whole cell inactivated vaccines—heat killed	Spray	Injection	70%	[19]
Extracellular product (ECP)—formalin treated	Intraperitneal	Intraperitneal	29%	[14]

**Table 3:** *Streptococcus agalactiae* immunogenic proteins in tilapia (*Oreochromis niloticus*).

Protein	Abbr.	Location	Ref.
LPXT motif of cell wall surface anchor family protein	LPTXG	Cell wall	[13]
Surface immunogenic protein	Sip	Cell wall	[12]
Truncated surface immunogenic protein	tSip	Cell wall	[22]
Fibrinogen binding protein	FbsA	Cell wall	[23]
Alpha-enolase (Enolase 1)	ENO1	Cell wall	[23]
Phosphoglycerate kinase	PGK	Cell wall	[24,25]
Ornithine carbamoyl-transferase	OCT	Cell wall	[24,25]
Extracellular products 89KDa protein	ECP89	Cell wall	[26]
Pyruvate kinase	PK	Cytoplasm	[25]
5′-endonucleotidase family protein	NT5C	Cell wall	[25]
Branched chain alpha-keto acid dehydrogenase subunit E2	BCKDC	Cytoplasm	[25]
Transketolase	TKT	Cytoplasm	[25]
Alpha-glycerophosphate oxidase	GIpO	Cytoplasm	[25]
Capsular polysaccharide protein E	CpsE	Cell wall	[27]
Immunogenic secreted protein	Isp	cell	[28]

**Table 4:** Subunit vaccines showing delivery systems and modalities of vaccination.

Vaccine	Vaccination	Challenge	RPS	Ref.
Subunit vaccines Sip (tSip (1))	Intraperitoneal	Intraperitoneal	55%	[22]
Subunit vaccines Ornithine carbamoyl-transferase	Intraperitoneal	Intraperitoneal	0%	[24]
Recombinant FbsA subunit	Intraperitoneal	Intraperitoneal	40.63%	[23]
Recombinant a-enolase subunit	Intraperitoneal	Intraperitoneal	62.50%	[23]
Subunit vaccines Phosphoglycerate kinase	Intraperitoneal	Intraperitoneal	35%	[24]
PMMMA-PLGA *	Oral ^1^	Bacterial inhibition test	ND	[34]

* Poly[(methyl methacrylate)-co-(methyl acrylate)-co-(methacrylic acid)]-poly(d,l-lactide-co-glycolide); ^1^ ND = not done.
